# Classifying publications from the clinical and translational science award program along the translational research spectrum: a machine learning approach

**DOI:** 10.1186/s12967-016-0992-8

**Published:** 2016-08-05

**Authors:** Alisa Surkis, Janice A. Hogle, Deborah DiazGranados, Joe D. Hunt, Paul E. Mazmanian, Emily Connors, Kate Westaby, Elizabeth C. Whipple, Trisha Adamus, Meridith Mueller, Yindalon Aphinyanaphongs

**Affiliations:** 1 Health Sciences Library, NYU School of Medicine, New York, USA; 2 Institute for Clinical and Translational Research, School of Medicine and Public Health, University of Wisconsin-Madison, Madison, USA; 3 School of Medicine, Virginia Commonwealth University, Richmond, USA; 4 Indiana Clinical and Translational Sciences Institute, Indiana University School of Medicine, Indianapolis, USA; 5 Clinical and Translational Science Institute, Medical College of Wisconsin, Milwaukee, USA; 6 Wisconsin Partnership Program, School of Medicine and Public Health, University of Wisconsin-Madison, Madison, USA; 7 Ruth Lilly Medical Library, Indiana University School of Medicine, Indianapolis, USA; 8 Ebling Library for the Health Sciences, School of Medicine and Public Health, University of Wisconsin-Madison, Madison, USA; 9 Population Health Sciences, School of Medicine and Public Health, University of Wisconsin-Madison, Madison, USA; 10 Department of Population Health, NYU School of Medicine, New York, USA

**Keywords:** Machine learning, Translational research, Knowledge translation, Text classification

## Abstract

**Background:**

Translational research is a key area of focus of the National Institutes of Health (NIH), as demonstrated by the substantial investment in the Clinical and Translational Science Award (CTSA) program. The goal of the CTSA program is to accelerate the translation of discoveries from the bench to the bedside and into communities. Different classification systems have been used to capture the spectrum of basic to clinical to population health research, with substantial differences in the number of categories and their definitions. Evaluation of the effectiveness of the CTSA program and of translational research in general is hampered by the lack of rigor in these definitions and their application. This study adds rigor to the classification process by creating a checklist to evaluate publications across the translational spectrum and operationalizes these classifications by building machine learning-based text classifiers to categorize these publications.

**Methods:**

Based on collaboratively developed definitions, we created a detailed checklist for categories along the translational spectrum from T0 to T4. We applied the checklist to CTSA-linked publications to construct a set of coded publications for use in training machine learning-based text classifiers to classify publications within these categories. The training sets combined T1/T2 and T3/T4 categories due to low frequency of these publication types compared to the frequency of T0 publications. We then compared classifier performance across different algorithms and feature sets and applied the classifiers to all publications in PubMed indexed to CTSA grants. To validate the algorithm, we manually classified the articles with the top 100 scores from each classifier.

**Results:**

The definitions and checklist facilitated classification and resulted in good inter-rater reliability for coding publications for the training set. Very good performance was achieved for the classifiers as represented by the area under the receiver operating curves (AUC), with an AUC of 0.94 for the T0 classifier, 0.84 for T1/T2, and 0.92 for T3/T4.

**Conclusions:**

The combination of definitions agreed upon by five CTSA hubs, a checklist that facilitates more uniform definition interpretation, and algorithms that perform well in classifying publications along the translational spectrum provide a basis for establishing and applying uniform definitions of translational research categories. The classification algorithms allow publication analyses that would not be feasible with manual classification, such as assessing the distribution and trends of publications across the CTSA network and comparing the categories of publications and their citations to assess knowledge transfer across the translational research spectrum.

**Electronic supplementary material:**

The online version of this article (doi:10.1186/s12967-016-0992-8) contains supplementary material, which is available to authorized users.

## Background

### Prior definitions

Since 2006, academic research centers functioning as National Institutes of Health (NIH) Clinical and Translational Science Award (CTSA) hubs have provided services, resources, and educational offerings to clinical and translational researchers. The objective of developing this infrastructure was to speed the movement of research findings from the lab to clinical studies and eventually to therapeutic practices that improve health in the community and reduce health disparities. Evaluation of these hubs and their programs presents many challenges, and a lack of consistency and clarity in definitions for key terms connected with CTSA goals is a core challenge in developing metrics of CTSA success. Evaluators from CTSA hubs have worked to develop definitions for these terms to assist in the complex challenges of assessing the impacts of the hubs on biomedical science, research, and workforce development, and on longer term objectives such as changes in clinical practices and improvements in health disparities and overall human health [1].

While the goal of the CTSA program is to speed the movement of research findings along the translational research spectrum, it is difficult to assess progress in this area due to the lack of clarity and consensus around the definitions of the stages of research along this spectrum. For more than a decade, different conceptualizations of the translational research spectrum have been proposed, with different ways of dividing up the spectrum and different definitions for the resulting categories [2–9]. These competing models create difficulties in communication around the translational research spectrum, and development of metrics based on conflicting models can lead to misinterpretations, particularly as translational researchers are typically unaware of competing definitions [10].

Recent recommendations from both the National Center for Advancing Translational Sciences (NCATS) [11] and the 2013 Institute of Medicine (IOM) report [12] classify translational research into five stages. While this agreement between the number of phases described by NCATS and the IOM report is a promising start for convergence, the respective models diverge significantly. Most significantly, the IOM report describes the T0 phase as basic science research [9], but defines this as including preclinical and animal studies, and so being squarely within the translational spectrum. The NCATS model also starts with “basic research” but places basic research outside of the translational spectrum and defines preclinical research as a separate stage. This marked lack of standardization impedes the broad utility of any work around publication classification.

Ultimately, translation is “the process of turning observations in the laboratory, clinic and community into interventions that improve the health of individuals and the public—from diagnostics and therapeutics to medical procedures and behavioral changes” [13]. Segmenting a process such as translational research into discrete categories inevitably involves a degree of arbitrariness and simplification. Despite these inherent shortcomings, agreement on definitions could pave the way for common metrics to distinguish between different components of this process and examine movement through the process.

### Prior approaches

Prior to the current conceptualization of the translational research spectrum, Narin broke the research spectrum into four categories from basic to clinical [14]. A number of efforts to look at the classification of journals and publications either used the Narin categories [14–16] or built on them [17, 18]. Approaches to categorization of publications often applied filters based on title words [16–18]. There have also been efforts to look at citation usage, or knowledge transfer, across categories [16, 19, 20]. More recently, Weber proposed a triangle of biomedicine [21] where articles are mapped to either humans, animals, or cells and molecules based on the medical subject headings (MeSH) used by PubMed. Weber also looked at the position of an article’s citations within the triangle of biomedicine to examine movement towards research on humans. However, these approaches are not directly applicable to classifying publications along the translational research spectrum, since they either ignore research beyond clinical or combine all human research into one category, rather than distinguishing between clinical research and public or population health research.

### Current challenges

All CTSA hubs are required to report publications that result from research that used CTSA services, resources, and educational offerings, or that received hub funding for pilot projects or career development (KL2 and TL1 awards). This stems, at least in part, from the requirement that publications comply with the NIH Public Access policy [22], and results in an extensive data set on which to base assessments of productivity, collaboration, and translation resulting from projects supported by CTSA hub resources. The importance of establishing metrics based on this data has been recognized both within and outside of the CTSA network [12, 23].

In its 2013 assessment of the CTSA program, the IOM report [12] stated that metrics need to move beyond the standard benchmark of counting publications, however it did not specify what types of publications-related metrics would be most useful. The IOM report also stated the importance of tracking research outcomes if the accountability of translational research is to be improved and recommended that CTSA hubs support research across the entire translational spectrum from T0 to T4. The challenge is that in order for hubs to assess whether they are in fact supporting the full range of translational research, they must have a means by which to classify research projects by their translational phase.

Current CTSA efforts around common metrics have focused on the Relative Citation Ratio [24], an article-level, field-normalized citation metric. This is a significant move beyond publication counts in that it actually begins to gauge the impact of CTSA supported publications rather than simply counting them. However, this metric still fails to address the translation of research findings. A publication and its citation network holds information about the nature of a research project, what that research is based on, and how the results of that research are being used. This information contains the promise of yielding information about translation, but exploiting it requires a scalable way to determine the translational phase of the research involved.

Bibliometric analysis of publications has the potential to contribute to a more in-depth understanding of how CTSA support moves research along the translational continuum and how that CTSA support ultimately contributes to improved health. Analysis of the types of translational research (T0 to T4) being supported and published by the CTSA program could help hubs track their relative success in achieving potential short, medium, and long term objectives, and allocate their scarce resources to maximize research necessary to advance translation.

### Our contribution

There are three significant challenges to measuring the impact of translational research publications. The first challenge is to establish a common set of definitions; without agreement on what translational research is, and what the progression of this research is, local measurements of impact will not be comparable to those from other CTSA hubs. The second challenge is to operationalize those definitions; consistent definitions without a consistent method for applying these definitions will also not produce comparable results. The final challenge is to create a methodology that facilitates the application of these definitions, so that categorizing publications or projects along the translational spectrum is sufficiently scalable to realistically allow organizations to implement these metrics given time and staffing constraints. In this study, we achieve consensus regarding definitions across five CTSA hubs, operationalize those definitions, and build classifiers to facilitate classification. We hope that this provides a strong basis for adoption of these definitions more broadly across the CTSA consortium.

We developed a checklist to produce greater consistency, or inter-rater reliability, in how we classified publications into our agreed upon categories of translational research. Sets of manually-classified publications were used to train machine learning-driven models to identify articles belonging to each translational research class. By using the categories from the 2013 IOM report [12] as a starting point, achieving agreement on definitions across the five participating CTSA institutions, and operationalizing these definitions, we believe we have laid the groundwork for better consistency across CTSA organizations in these definitions. The development of consistency across the CTSA hubs is important to the consortium, as evidenced by the work on developing common metrics and that consistency enables measurement that can inform the effective allocation of resources, identification of institutional strengths, and return on investment measurements within and across CTSA hubs.

Our work differs from previous efforts to automate categorization of articles in this area by using the most current conceptualization of the translational research spectrum. The two most significant ways in which the translational research spectrum varies from other categorization schemes is in its inclusion of post-clinical research (e.g. health services research, patient-centered outcomes research, comparative effectiveness research), and the inclusion of human research that studies underlying disease mechanisms rather than testing clinical interventions in the T0 category. Our use of the machine learning approach is also a departure from previous research classification models.

## Methods

Part 1 of the methods details the process used by the collaborating sites to arrive at agreement on the definitions of categories along the translational research spectrum and to develop a checklist to improve inter-rater reliability. Part 2 details the methods used in the construction of the training set, document processing, application of machine learning algorithms to the training set, and internal and external performance estimation.

### Part 1: translational category definition and checklist development

To arrive at the final criteria for manual coding of publications into categories along the translational research spectrum, one or more representatives from each of the collaborating institutions engaged in an iterative process of consensus meetings, definition development, and coding. We based our initial categorization scheme on the definitions used by each CTSA institution, if applicable. Those definitions were drawn from reviews of the literature [6, 8, 9, 12, 25] and from the work of other CTSA hubs (Harvard [26], University of Texas-Southwestern Medical Center [27], University of Wisconsin, Indiana University, University of Pittsburgh). Next, publication coders participated in several meetings to develop a shared mental model of how to define the research spectrum prior to developing the training set.

After agreement on definitions for each of the categories of translational research, three pilot rounds were performed in which small sets of publications were manually coded by all available coders, with at least one coder from all participating institutions. The number of coded sets for each round varied between seven and 10 as not all coders were available at all times for each institution, and at each institution, multiple coders might produce either multiple individual sets or a single collaborative set of coded publications. The first pilot set was constructed by choosing publications intended to highlight issues within the definitions or different interpretations of the definitions by individual coders. The second pilot set was randomly selected from publications that acknowledged the CTSA grants of each of the five collaborating institutions. The third pilot set was a combination of randomly selected publications and publications selected from the first two pilot sets. These coding pilots were undertaken to test inter-rater reliability and determine the need for further refinement of definitions.

Poor inter-rater reliability resulted from coding the pilot projects using the definitions, with agreement for the three sets of articles ranging from 0 to 14 %. The large number of coders increased the possibility of disagreement, therefore, we also assessed reliability by taking into consideration articles on which all but one coder agreed, for which the maximum percent agreement was 25. The poor inter-rater reliability led us to develop a checklist for each of the categories along the translational spectrum. An iterative process similar to that employed for definition development allowed us to reach consensus on the checklist.

### Part 2: translational category machine learning models

#### Corpus construction

To train the classifiers, the five categories of translational research were grouped into three categories, roughly corresponding to basic, clinical, and post-clinical research. T3 and T4 articles were grouped into one set, due to the low frequency of T4 articles during initial piloting of classification schemes. Similarly, T1 and T2 articles were grouped into one set due to the low frequency of both types of articles in the training set. An additional category was designated as TX, denoting articles that did not fall along the translational research spectrum, although they cited a CTSA hub. The training set for each classifier would then consist of articles that were coded as being in a particular category and those that were coded as not being in that category, the latter set including TX for each of the categories.

The training sets were assembled in two stages. In the first stage, 200 publications were selected as follows: Using the Entrez E-utilities from NCBI [28] within Matlab [29] code, we accessed PubMed IDs (PMIDs) for all publications indexed in PubMed to past or present CTSA award grant numbers for collaborating institutions. Forty publications from each institution were randomly selected from the subset containing abstracts. This was done using the randperm function in Matlab to create a random permutation of the indexes into the list of PMIDs and stepping through the randomized indexes into the list of PMIDs from each and selecting the first 40 that had an abstract. Each institution was assigned 80 publications to manually code: their own 40 publications, plus 10 publications from each of the other four institutions. The publications were assigned so that each of the 200 publications was coded by two institutions. Within each institution, each publication was coded by between one and three coders. For each publication, coders assigned both a category from the translational spectrum and the number of the checklist item(s) that led them to choose that code.

After the initial coding, the following process was used to select the training set. Publications for which there was agreement between the two coding institutions were included in the training set. For publications where the codes were not in agreement, the lead author acted as a third coder and looked at the selected codes, the checklist items, and the article features to determine a code. When that code agreed with one of the institutions, it was assigned to the article, which was then included in the training set. When the lead author’s code did not agree with the codes of either institution, the article was excluded from the training set.

Due to a low frequency of T1 and T2 articles in the initial corpus, a second set of articles was assembled for coding. Filtering the articles by a set of search terms was used to increase the frequency of T1 and T2 articles within the training set. A set of highly sensitive search terms (Additional file 1) was developed such that all previously identified T1 and T2 articles were retrieved using these terms. These search terms produced a large percentage of articles that were not coded as T1 or T2. This large number of false negatives was consistent with the goal of increasing the percentage of T1 and T2 articles in the training set, while not limiting the range of T1 and T2 articles represented. Articles were again randomly selected from publications indexed to the award numbers for the five participating CTSA institutions, using the Entrez E-utilities from NCBI within Matlab code. Articles included in this training set were limited to those meeting three criteria in addition to the grant number indexing: (1) articles with an abstract, (2) articles not included in the first training set, and (3) articles with one of the T1/T2 search terms in the title and/or abstract. Not all of the five institutions had 40 articles that met these criteria, therefore the second training set consisted of only 186 publications.

#### Document preprocessing

Document preprocessing converts the text of the scientific articles into a matrix that can be processed by the machine learning algorithms. We used a “bag of words representation” where the text is represented by the set of words without regard to grammar or word order, with words represented by the document zones where they occur [30]. Those zones included the title, abstract, and the full MeSH term/phrase. This encoding produces a matrix where the rows are individual documents and the columns are individual words in either the title, abstract, or mesh terms.

Each individual cell contains an importance weight that indicates the usefulness of the word for classification. The assumption is that words that have high frequency in one document are more discriminatory than words that appear across many documents. The classic algorithm for weighting the importance of a word is term frequency—inverse document frequency [31]; we use logarithmic frequencies with redundancy normalized by the square root of the sum of the squares of the individual word weights, i.e., the L2 norm. This algorithm shows superior performance in benchmark work [32].

Finally, in contrast to other work in text classification, we did not remove stop words, i.e., common words such as “and” or “the,” nor did we stem the words, i.e., replace all forms of a word with the root word, for example “smoking” and “smoked” are mapped to “smok*”). While we consider these feature engineering approaches to be refinements toward improving performance, in our experience, these approaches will have a minor impact on performance.

#### Machine learning algorithms

Because the algorithm that will perform best for any given classification problem is not known a priori, we relied on prior benchmarks in text classification to guide our choices for candidate algorithms. Specifically, we chose naïve Bayes as a standard baseline algorithm in text classification to compare to three of the highest performing algorithms in multiple benchmarks: Bayesian logistic regression, random forests, and support vector machines [33].

##### Naïve Bayes

This algorithm directly applies Bayes theorem to the classification task and assumes that the probability distribution of a feature is independent of another feature, given the class labels. We used the Multinomial Naïve Bayes [34] implementation in the mallet package [35]. This algorithm does not require tuning.

##### Bayesian logistic regression

We employed Bayesian logistic regression because this algorithm demonstrated superior performance in text classification benchmarks. This algorithm constrains the coefficients using a Laplace prior and allows an efficient solution to the convex optimization. We used the bbrtrain [36] implementation for this study. We used the autosearch option to optimize the regularization parameter. This option does a grid search using tenfold cross validation across the lambda parameters of 0.01–316 in multiples of the square root of 10.

##### Random forests

We employed the random forest implementation in the fest [37] program. Random forests [38] are an ensemble classification method. The method produces a classification tree at each iteration. This classification tree is built from a random subset of the data, and at each node in the tree, a random subset of predictor variables are selected. Multiple trees are constructed in this fashion until the classification of these individual trees are combined to form a final prediction at test time. We considered tuning parameters of a forest of 100, 300, and 500 trees and feature selection at each node of square root of the number of features ×1, ×2, and ×3.

##### Support vector machines

We employed a linear support vector machine (SVM) classification algorithm as implemented in the liblinear package [39]. The linear SVMs calculate maximal margin hyperplane(s) separating the two classes of the data. These linear SVMs demonstrated superior text classification performance compared to other methods [40]. The liblinear implementation is a version of the support vector machine optimized for quickly finding a linear separating hyperplane. We used liblinear as implemented in libSVM v1.96 [41] with costs of 0.1, 1.0, and 10 and differential weights for costs according to the prior category distribution of the training set.

#### Performance estimation (internal)

##### Nested stratified cross validation

The goal of our algorithms is to predict translational class in a future test set. We simulated this prediction with an unbiased estimate using a nested cross validation protocol [42]. This protocol divides the data into a training set, a validation set, and a test set. The training set is used to build the model. The validation set is used to optimize any parameters of the machine learning algorithm. Finally the test set is used to estimate performance of the built model. We employed fivefold cross validation. The first three folds are used to train the model. The 4th fold is the validation set. The 5th fold is the test set. We executed this procedure five times using each fold as the test set once and reported the average of the five times. Finally we repeated each run 10 times and report the average result over the 10 runs. The folds are also stratified to maintain the proportion of documents in each classification stage.

##### Performance metrics

Results are reported as area under the (receiver operating) curve (AUC). We employed this reporting metric because it is commonly used to evaluate classification models, is invariant to the imbalanced prior probabilities of each translational class, and shows performance across the entire spectrum of possible sensitivities and specificities. The area under the receiver operating curve ranges from 0.5 to 1.0. The score corresponds to the probability that a document in the translational class will rank higher than a document not in the translational class. A score of 1.0 means that 100 % of the time, the algorithm ranks a translational class document higher than a document not in the translational class.

#### Performance estimation (external)

We did an additional external validation to evaluate generalizability of the built models. We applied the models we built in each translational class to a set of articles indexed to CTSA grants in PubMed.

##### Corpus construction

We downloaded 40,633 articles indexed in PubMed to 134 active and inactive CTSA center and training grants from 61 institutions as of 8/12/2015. We defined this “external test set” as the set that included all articles indexed to those grants except for those that were in the training set and those that did not have an abstract. All articles were scored by each of the three classifiers (T0, T1/T2, T3/T4) (scores in Additional file 2). A corpus was constructed of the 100 articles with the highest scores from each of the classifiers, so the total corpus was 300 articles, and each article was coded by two institutions. Articles were randomly assigned to each of the five collaborating institutions for coding, so each institution was assigned 120 articles to code. The coders were blinded to the scores; articles assigned to each institution were ordered by PMIDs so that the articles from each of the 3 categories were interspersed. When the two institutions did not agree on the coding, the lead author used the article information and the two coder’s checklist codes to make a determination on article classification, i.e., acted as a third coder. The PubMed IDs and coding for the articles used for the validation are available in Additional file 3.

##### Final model construction

We built the final machine learning filter models to apply to the external test set using the highest performing classifier, Bayesian logistic regression. The algorithm takes settings (also called hyperparameters) that control how closely the algorithm will fit the data. These settings were chosen using the “autosearch” capability of bbrtrain. Autosearch divides the data into tenfold. The method implements a search across the various settings and finds the setting that maximizes the sum of the log-likelihoods. We then built the final model using that setting.

##### Performance metrics

We used a simple measure of accuracy within the top 100 lists.

## Results

### Part 1

#### Definition and checklist consensus

The iterative process of coding articles and revising the definitions, resulted in resolution of several key points of disagreement regarding the definitions of each of the phases of translational research. Agreement was reached by moving away from generalizations about types of research, such as “T0 is animal research” or “systematic reviews are T3,” and looking at the intent of the research and how its outcomes could be used.

The point of greatest disagreement was whether research on humans could be categorized as T0 research. Views on this ranged from no inclusion of human data in T0, to inclusion of human research only if it used existing data, to inclusion of human studies that explored underlying mechanisms as long as there was no intervention. We ultimately agreed that human research fits the definition of T0 research when the focus of that research is to elucidate biological, social, and behavioral mechanisms that underlie either health or disease; human research only falls into the T1 category when it focuses on applying that understanding of the system to a health application.

The categorization of health services research was a second point of contention. Although some health services research is interventional, other studies identify issues or explore underlying mechanisms of health services delivery without testing solutions. While there was agreement that interventional health services research should be categorized as T3, some felt that research to elucidate the process should fall under T0, while others felt that any health services research should be categorized as T3. We were able to agree that health services research falls under T3, whether or not the study involved an intervention by distinguishing between elucidating mechanisms underlying health care delivery and elucidating mechanisms underlying disease and health.

A third issue was the categorization of systematic review articles; some coders thought that systematic reviews should automatically fall within the T3 category and others argued that this should be dependent on the topic of the review or the intent of the research under review. The resolution of this issue was to clarify that unless the systematic review had the potential to lead to a practice guideline, it would not fall into the T3 category.

The full definitions arrived at through this process are shown in Table 1.

**Table 1:** Full definitions for each of the phases along the translational research spectrum

T0	*Basic biomedical research: identification of opportunities and approaches to health problems*
	Includes preclinical and animal studies
	May or may not consider a particular disease process
	May include human subjects, but does *not* include interventions with human subjects
	Goal is to understand the human condition and environment as it exists
	Focuses on understanding biological, social and behavioral mechanisms that underlie health or disease
	Defining mechanisms, biomarkers, targets for therapeutic development; drug discovery (lead molecule screening, optimization, formulation); prototyping; physical assessments (radiology, laboratory, biopsy)
	Can include non-interventional, correlational epidemiologic studies using existing large data sets
	Studies mechanisms or derive modifications of cells, proteins, and DNA present in human disease processes
	Identifies functional significance and mechanisms of genomic polymorphisms identified by human genome-wide association studies
T1	*Translation to humans: seeks to move fundamental discovery into health application; provide clinical insights*
	Involves proof of concept studies
	Includes Phase 1 clinical trials
	Healthy subjects or select population of patients
	Small sample size
	Tests for safety
	Focuses on new methods of diagnosis, treatment, and prevention
	Takes place in highly controlled research settings
T2	*Translation to patients: health application to implications for evidence-based practice guidelines*
	Involves controlled clinical research studies which may lead to the basis for clinical application and evidence-based guidelines
	Yields knowledge about the efficacy of interventions in highly-controlled/protocol-driven settings
	Goal is to identify and analyze the optimal effects of an intervention on the human condition or environment
	Phase 2 clinical trials—focus on safety and efficacy (dose-response)
	Select population of patients
	Relatively large sample size
	Phase 3 clinical trials—focus on safety and efficacy
	Select population of patients
	Special groups of patients (ex. renal failure)
T3	*Translation to practice: practice guidelines to health practices*
	Includes comparative effectiveness, pragmatic clinical trials, community based participatory research, dissemination and implementation research, and clinical outcomes research, post-marketing analysis (Phase 4)
	Health services research, including reasons for gaps in care and delivery of recommended and timely care to the right patient
	Meta-analyses, and systematic reviews involving interventions
	Development and implementation of evidenced-based guidelines, policies, and best practices
T4	*Translation to communities: health practice to population health impact, providing communities with the optimal intervention*
	Includes population-level outcomes research: population monitoring of morbidity, mortality, benefits, and risks
	Focuses on wider dissemination/implementation of improved practices/interventions (taking to scale)
	Focuses on impacts of policy and/or environmental change
	Studies focusing on disease prevention through lifestyle and behavioral modifications
	Documents “real-world” health outcomes of population health practices associated with improved disease prevention and reduced medical costs
	Results in true benefit to society

The problem of poor inter-rater reliability seen when coding the sets of pilot articles was resolved by introducing checklists for each of the categories of definitions. Using the checklist, shown in Table 2, agreement across all coders increased from 14 to 31 % and agreement across all but one coder increased from 25 to 67 %. Because of this marked increase in inter-rater reliability, the checklists, rather than the full definitions, were used by the coders when classifying publications for the training set.

**Table 2:** Checklist for each of five categories along the translational research spectrum

1. Does the research involve use of animals?	T0
2. Does the research involve study of mechanisms, relationships, or modification of proteins, DNA, or cells?	T0
3. Is the research a Genome Wide Association Study, determining association of a SNP with a particular disease state?	T0
4. Does the study examine drug interactions with molecular receptor or enzyme; effects on cell biochemistry, or how to optimize interaction?	T0
5. Does the publication describe the creation of a prototype for a new medical device?	T0
6. Does the research test a new methodology with potential for use in diagnosis, treatment, or prevention, providing a basis for follow-up study to directly test the methodology for safety, feasibility, preliminary results, etc. NOTE: This may be done through use of existing EHR data, other health datasets, or through taking measurements from humans, such as physical assessments (radiology, laboratory, biopsy) or response to stimulus (but NOT response to intervention).	T0
7. Is a new association between biological, social, and/or behavioral states determined, including association between presence or progression of a disease state and a biomarker, social, or behavioral state? Note: This may be done through use of existing EHR data, other health datasets, or through taking measurements from humans, such as physical assessments or response to stimulus (but NOT response to intervention).	T0
8. Does the research explore a biological, social, or behavioral mechanism, including the mechanism underlying the presence or progression of a disease? NOTE: This may be done through use of existing EHR data, other health datasets, or through taking measurements from humans, such as physical assessments or response to stimulus (but NOT intervention).	T0
9. Is it a systematic review or meta-analysis of research that seeks to establish a correlation or elucidate a mechanism (i.e., review of T0 research), or to establish need for further work at the T0 level (e.g., further methodology development)?	T0
10. Is it a Phase I clinical trial?	T1
11. Does the study test the effect of a new intervention on healthy volunteers in a controlled clinical setting?	T1
12. Does the research suggest a new method of diagnosis (e.g., biomarker) or new intervention, determine feasibility or safety, or test it in a small group? Note: This should not be research that would lead directly to a practice guideline.	T1
13. Does research describe the use of a new device on a small population to determine potential usefulness and usability?	T1
14. Is it a Phase II or Phase III clinical trial?	T2
15. Does the study test efficacy and/or determine dosing levels of an intervention in a population with a given disease in a controlled clinical setting?	T2
16. Does the study determine the optimal use of a new medical device?	T2
17. Does the study determine the efficacy or optimal use of a new method of diagnosis or prevention, including through the use of existing datasets?	T2
18. Is it a Phase IV clinical trial?	T3
19. Does the research study effectiveness of an intervention or method of diagnosis in the clinic or community, either through the use of existing health datasets or new research?	T3
20. Does the research study inconsistency or variation in the application of a diagnosis or intervention?	T3
21. Does the research compare the effectiveness of existing health care interventions to determine which work best for which patients and which pose the greatest benefits and harms?	T3
22. Does the research involve interventions in the community with input from community members?	T3
23. Does the research determine mechanisms underlying effective health care delivery in practice or community settings?	T3
24. Does the research identify problems with effective health care delivery in practice or community settings?	T3
25. Does the research test an intervention to improve healthcare delivery in practice or community settings?	T3
26. Does the research study real world factors affecting interventions (cost, convenience, accessibility, patient preferences)?	T3
27. Does the research determine reasons why gaps in care exist?	T3
28. Is it a systematic review or meta-analysis of interventions, diagnoses, or something that could lead directly to practice guidelines, or a review article that suggests practice guidelines or workflow?	T3
29. Does the publication provide evidence-based guidelines/policies or best practices?	T3
30. Does the research use large datasets to monitor morbidity, mortality, benefits, or risks of interventions in populations?	T4
31. Does the research study the incidence or prevalence of a disease in a population?	T4
32. Does the research examine problems with, mechanisms underlying, potential interventions, impact, or real world outcomes (e.g., level of disease prevention, reduced medical costs) of population level practices/interventions?	T4
33. Does the research study the impact on health of a policy or environmental change that affects a population?	T4
34. Does the research focus on the development or outcomes of population level behavioral/lifestyle interventions?	T4

The full definitions were of limited utility for coding due to the poor inter-rater reliability achieved with the definitions alone and were seen as too detailed for the purpose of communicating the general ideas underlying the definitions. Hence, a concise set of definitions, shown in Table 3, were developed using the same consensus building process as for the detailed definitions. The intent of the concise definitions was to convey to a general audience the core principles behind the definitions for each of the categories of translational research.

**Table 3:** Concise definitions for each of the phases along the translational research spectrum

T0	***Basic biomedical research***
	*Identification of opportunities and approaches to health problems*
	Includes: preclinical and animal studies; GWAS studies; studies of cells, proteins, and DNA; studies on humans or existing datasets that focus on understanding biological, social and behavioral mechanisms that underlie health or disease
T1	***Translation to humans***
	*Seeks to move fundamental discovery into health application; provide clinical insights*
	Includes: proof of concept studies; Phase 1 clinical trials; studies testing feasibility or safety of a new method of diagnosis, treatment, or prevention
T2	***Translation to patients***
	*Health application to implications for evidence-based practice guidelines*
	Includes: Phase 2/3 clinical trials; studies to test efficacy of interventions in highly controlled settings
T3	***Translation to practice***
	*Practice guidelines to health practices*
	Includes: Phase 4 clinical trials, comparative effectiveness research, community based participatory research, dissemination and implementation research, clinical outcomes research, health services research, meta-analyses/systematic reviews of interventions, development/implementation of guidelines
T4	***Translation to communities***
	*Health practice to population health impact, providing communities with the optimal intervention*
	Includes: population-level outcomes research; wider implementation and dissemination; policy impacts; disease prevention through lifestyle/behavior modifications; real-world health outcomes; true benefit to society

#### Checklist application

The training set was constructed with subsets of articles from both the initial 200 randomly selected articles and from the second set of articles filtered to increase the incidence of T1/T2. Of the initial 200 articles, 185 were included in the training set, with one article coded as belonging to both T0 and T1, resulting in 186 codings. Of the second set of 186 articles, 164 were included in the training set. Of the 186 articles, 25 % (46) were either T1 or T2, compared to 9 % (18) of the 200 articles in the first training set. The total number of publications included in the training set was 349, but there were 350 codings due to the one article coded as both T0 and T1. The full breakdown of coding is shown in Table 4. All PubMed IDs and codes for the training set are available in Additional file 4.

**Table 4:** Breakdown of training sets by categories into which articles were classified by coders

	Training set 1	Training set 2	Combined training set
T0	106	56	162
T1/T2	18	46	68
T3/T4	44	50	94
TX	18	12	30
Total included in training set	*186*	*164*	*350*
Not included in training set	15	22	33
Total	*201*	*186*	*387*

T0 through T4 are the phases of research along the translational spectrum. TX denotes publications that were determined by the coders to not fall into any of the T0 through T4 categories. Uncoded denotes publications on which no agreement could be reached by the coders as to the correct category. Note that there is one article that was determined to fall into both the T0 and T1 categories, thus resulting in a total of 387 codings for the 386 articles that were coded

The coding for the combined training set of 386 documents had an inter-rater reliability of 64 %, yielding 139 publications on which the coders from two institutions disagreed. Because some institutions had multiple coders, this might involve disagreement between anywhere from two to five coders. Of the 139 publications without initial agreement, 17 had disagreement across three or more categories. Of those 139 articles, 103 were resolved through having an additional coder classify the publication. The most common discrepancies involved the T0 category. Of the 139 articles, 34 involved a disagreement between a classification as T0 and a classification as T1/2, and an additional 33 involved disagreement between T0 and T3/T4. TX represented a determination by a coder that the article did not fall on the translational research spectrum, and 32 of the 139 articles involved at least one coder specifying TX to indicate no classification and one or more coders placing the article somewhere on the translational research spectrum.

### Part 2

#### Performance estimation (internal)

The results in Table 5 demonstrate that classifiers built in each translational class are highly discriminatory. The T0 classifier performs with a maximal AUC of 0.94 with random forests and linear support vector machines. The T1/T2 classifier performs with maximal AUC of 0.84 with random forests, Bayesian logistic regression, and linear support vector machines. Finally the T3/T4 classifier performs with a maximal AUC of 0.92 for Bayesian logistic regression and linear support vector machines.

**Table 5:** AUC and performance ranges for each classifier with different machine learning algorithms

Classifier	Translational class		
	T0	T1/T2	T3/T4
Naïve Bayes	0.91 (0.80–0.97)	0.78 (0.45–0.97)	0.87 (0.72–0.97)
Liblinear (linear support vector machine)	*0.94 (0.93–0.96)*	*0.84 (0.76–0.98)*	*0.92 (0.90–0.94)*
Random forest	*0.94 (0.84–0.98)*	*0.84 (0.75–0.98)*	0.87 (0.72–0.98)
Bayesian logistic regression	0.92 (0.82*–*0.99)	*0.84 (0.53–0.99)*	*0.92 (0.82–0.99)*

Best performing algorithm(s) for each classifier are italicized

The performance ranges have variability across the cross validation folds with Bayesian logistic regression illustrating a wide range of performance. Also, as expected, the Naïve Bayes classifier performs the worst. Naïve Bayes assumes conditionally independent features given the class and as in most text classification tasks, this assumption hurts classifier performance. However, for the T0 classifier, the loss of performance is limited; possibly because individual words in the T0 class are highly predictive of class.

#### Performance estimation (external)

In the results shown in Table 5, we internally validated the models using a stratified N-fold cross validation procedure. The stratified N-fold cross validation procedure makes two assumptions. First, the procedure assumes the word/token distributions are from the same distribution. By design, the cross validation makes this assumption as training and testing documents are randomly chosen from the dataset. Second, the procedure assumes that the proportion of articles in each class is higher than in actual data. Recall, the training datasets were built with a case–control design of identifying positive articles for each class and then considering all other articles not in the positive class as negative. These artificial priors typically overestimate generalization performance. Applying the models to an external dataset would validate the procedure in real world datasets where the proportions of articles in each class are vastly different.

We built final models with the labeled document collections and applied these models to unlabeled documents attributed to CTSA grants. We then manually coded the top 100 documents scored by each of the three classifiers, e.g., T0, T1/T2, T3/T4. Category agreement was 99, 91, and 95 respectively.

## Discussion

### Part 1

#### Definition and checklist consensus

The goal of the definitions and the checklist is to clearly define coherent and distinct categories that present as clear a path as possible through the translational research spectrum for as many types of translational research as possible. Interventional research produces the clearest path along the translational research spectrum as it goes through clinical trial phases that fall clearly into the T categories, while other paths presented more of a challenge as they are less distinctly defined. The questions of coherence and distinctness are most clearly raised by T0 research. T0 research encompasses a very broad range of research types and includes certain types of human research, potentially rendering it too similar to T1/T2 research. Our machine learning approach addressed these issues as the good classification seen would not be possible with categories that are not sufficiently coherent and distinct from one another.

#### Checklist application

The degree of inter-rater reliability seen reflects both the improvements in definition interpretation seen with the introduction of the checklist, as well the the significant room for disagreements in interpretation that still exists. While it is unlikely that all disagreements across coders can be eliminated given the varied and complex nature of translational research, the high percentage of articles that required a third coder for correct classification suggests that improvements in the checklist and/or increased coder experience may lead to greater inter-rater reliability.

#### Limitations

While a strength of this study is that the definitions and checklist were developed with input from five CTSA institutions, this still constitutes less than ten percent of CTSA hubs, so the definitions and checklist may not reflect the perspective of the broader CTSA consortium. This limitation was mitigated by the participation in the collaboration of two former chairs of the CTSA Consortium Evaluation Key Function Committee Definitions Workgroup, who brought a valuable perspective from their extensive work around definitions with many of the CTSA institutions.

Another limitation is that the checklist was applied by coders having a variety of backgrounds, and hence a diverse understanding of the different topic areas of coded publications. This was mitigated by engaging in extensive piloting, by having developed the checklist through consensus meetings, and by having each publication coded by two institutions and using a third coder to arbitrate disagreements.

### Part 2

#### Performance estimation (internal)

The high discriminatory performance suggests several conclusions about the process of classification and the content composition of the classes. First the labeling process is reliable. Poor rater reliability in labeling would result in poor performing classifiers. These results suggest the opposite. The labeling process is reliable and high performing classifiers were built. Second, the content for each class is coherent. Highly incoherent words and tokens in the title and abstracts of the content would result in poor performing classifiers as the classifiers would not be able to identify words and tokens that can discriminate. These results suggest that the content itself is highly coherent. For example, in the T1/T2 class, the presence of the tokens “randomized controlled trial” is highly coherent in articles of this topical class and these tokens do not appear highly in the other classes.

A more thorough analysis of the features selected, the false positives and false negatives incurred in the training sets, and stability of the models from the composition of the training sets are all points for future research.

#### Performance estimation (external)

Our models performed as expected in identifying the translational class of publications with high discrimination. The method of performance estimation of looking at the top 100 articles was selected to address a driving use case of classifying a large number of articles for a given CTSA hub. For this use case, it would be expected that manual coding would still be done for low scoring articles, but that the classifiers would minimize the work of manual coding by eliminating the need to code high scoring articles. The high degree of accuracy seen for these high scoring articles supports the usefulness of these classifiers for this purpose.

#### Limitations

##### Model stability over time

Topics in each class may change over time and may require periodic updating of the models. Models will eventually become obsolete. However, the key term here is eventually. It is easy to suggest examples where the model drift may occur. In an extreme example, if the models are not built with more recent data, then certain classifications will be missed. For example, if models were built using articles before the widespread use of genetic information in T0 studies, applying the models to future data will not identify T0 studies that use genetic information. Likewise, examples of the models remaining relevant are also conceivable. For example, the randomized controlled trial is an acceptable model for T1/T2 articles and this gold standard will likely not change. Classifiers that we have built for other purposes have demonstrated stability over time [43]. However, in this study, we consider our results preliminary and will require additional labels, validation, and verification to understand their classification behavior over time.

##### Training set selection bias

Aside from model drift, another potential source of model bias is topical content specific to the universities chosen for this pilot. For example, one or more institutions may nationally lead in T3/T4 cardiology research and the models will identify articles of this type correctly. However other institutions may do cardiology research but not in the T3/T4 classes, and the model will incorrectly assign weight to this content. While future work is planned to build training sets without this limitation, the magnitude of this limitation was small enough to still produce good classifiers as indicated by the high AUC values.

##### Artificial to natural priors

We used artificial priors and a case control design in building our training sets. The full extent of how using artificial priors will affect the classification performance, calibration, and generalization of the models requires further exploration.

##### Performance/thresholding considerations

A key element in applying these models is model calibration and acceptable false positives rates for specific use cases. The classification algorithms will return a score but these scores are not necessarily probabilities. The scores require calibration to probabilities. In Table 6, we show one experiment that shows preliminary evidence that the scores/probabilities produced by Bayesian logistic regression are fairly well calibrated.

**Table 6:** Results for coding by lead author of 50 articles randomly selected from each decile of T0 classifier scores (PubMed IDs and coding available in Additional file 5)

Decile	T0	notT0	Threshold	FPR	TPR
0.9–1.0	46	4	1	0	0
0.8–0.9	40	10	0.9	0.013986	0.214953
0.7–0.8	36	14	0.8	0.048951	0.401869
0.6–0.7	32	18	0.7	0.097902	0.570093
0.5–0.6	22	28	0.6	0.160839	0.719626
0.4–0.5	12	38	0.5	0.258741	0.82243
0.3–0.4	12	38	0.4	0.391608	0.878505
0.2–0.3	8	42	0.3	0.524476	0.934579
0.1–0.2	5	45	0.2	0.671329	0.971963
0–0.1	1	49	0.1	0.828671	0.995327

Columns contain the number of publications classified as either T0 or not T0 above each threshold classifier value, along with the calculated false positive rate (FPR) and true positive rate (TPR)

As Table 6 shows, the probability produced by Bayesian logistic regression is well calibrated in the T0 class. The intercept is −0.09 which indicates that the predicted probabilities are slightly lower than expected, and the slope is 1.0 indicating good calibration.

##### Error analysis

More iterative refinement of the features is possible. A thorough error analysis of false positive and false negatives may reveal additional features or techniques to improve classification performance. Further refinement and error exploration is open for future research.

## Conclusions

The two-fold results of this study support the use of the definitions of categories along the translational spectrum provided here by the broader CTSA consortium and translational research community. The agreement across five CTSA institutions on detailed definitions provides a good basis for broader uptake. The success of the machine-learned classifiers supports the assertion that the category definitions arrived at are both coherent and distinct from each other. The classifiers also operationalize the definitions in a way that makes metrics dependent on publication classification feasible. Further work is needed to build a training set that is drawn from a broader pool of articles and is larger to both eliminate potential bias in the classifiers and to determine if it is possible to build classifiers with good performance for each of the five categories. Additional improvements in classification may be possible with improved feature engineering (i.e. concept detection, non bag-of-words methods) or algorithm improvements using deep learning or sequence based classifiers. Nevertheless, even these preliminary results provide a strong basis for adopting these definitions across the CTSA consortium and using these classifiers to assess the distribution of CTSA hub research output across the translational research spectrum. Because these classifiers provide needed infrastructure to begin to assess how research moves across the translational spectrum, they also provide a basis for developing metrics to assess CTSA impact on that movement.

## Additional files

10.1186/s12967-016-0992-8 Filter terms: Search terms used to filter randomly selected articles indexed to CTSA grants in order to increase the incidence of T1 and T2 articles in the training set.
10.1186/s12967-016-0992-8 CTSA article scores: PMIDs for 40634 articles indexed to CTSA grants in PubMed with scores from each of the three classifiers for each PMID.
10.1186/s12967-016-0992-8 External validation: For each of 300 PMIDs (top 100 scored from each classifier), the file lists the classifier for which it received a top score, the classification given by the two coders, and, when applicable, the decision of the third coder.
10.1186/s12967-016-0992-8 Training set: For each PMID in training set, the file lists the manual codings and, where applicable, the decision of the lead author.
10.1186/s12967-016-0992-8 Threshold validation: For 50 articles randomly chosen from each decile of the scores returned by the T0 classifier this file lists the PMID, the classifier score, and a manual classification of either “T0” or “not T0”.

## Abbreviations

NIH: National Institutes of Health
CTSA: Clinical and Translational Science Award
NCATS: National Center for Advancing Translational Sciences
IOM: Institute of Medicine
AUC: area under the receiver operating curves
MeSH: medical subject headings
PMID: PubMed Identifiers
SVM: support vector machine
FPR: false positive rate
TPR: true positive rate
